# Smell Detection Agent Optimisation Framework and Systems Biology Approach to Detect Dys-Regulated Subnetwork in Cancer Data

**DOI:** 10.3390/biom12010037

**Published:** 2021-12-27

**Authors:** Suma L. Sivan, Vinod Chandra S. Sukumara Pillai

**Affiliations:** 1 Department of Computational Biology and Bioinformatics, University of Kerala, Trivandrum 695581, India; 2 Department of Computer Science, University of Kerala, Trivandrum 695581, India; vinodchandrass@gmail.com

**Keywords:** differential expression, subnetwork, topological weight, smell detection agent optimisation, breast cancer, colorectal cancer, disease genes, drug target

## Abstract

Network biology has become a key tool in unravelling the mechanisms of complex diseases. Detecting dys-regulated subnetworks from molecular networks is a task that needs efficient computational methods. In this work, we constructed an integrated network using gene interaction data as well as protein–protein interaction data of differentially expressed genes derived from the microarray gene expression data. We considered the level of differential expression as well as the topological weight of proteins in interaction network to quantify dys-regulation. Then, a nature-inspired Smell Detection Agent (SDA) optimisation algorithm is designed with multiple agents traversing through various paths in the network. Finally, the algorithm provides a maximum weighted module as the optimum dys-regulated subnetwork. The analysis is performed for samples of triple-negative breast cancer as well as colorectal cancer. Biological significance analysis of module genes is also done to validate the results. The breast cancer subnetwork is found to contain (i) valid biomarkers including *PIK3CA*, *PTEN*, *BRCA1*, *AR* and *EGFR*; (ii) validated drug targets *TOP2A*, *CDK4*, *HDAC1*, *IL6*, *BRCA1*, *HSP90AA1* and *AR*; (iii) synergistic drug targets *EGFR* and *BIRC5*. Moreover, based on the weight values assigned to nodes in the subnetwork, *PLK1*, *CTNNB1*, *IGF1*, *AURKA*, *PCNA*, *HSPA4* and *GAPDH* are proposed as drug targets for further studies. For colorectal cancer module, the analysis revealed the occurrence of approved drug targets *TYMS*, *TOP1*, *BRAF* and *EGFR*. Considering the higher weight values, *HSP90AA1*, *CCNB1*, *AKT1* and *CXCL8* are proposed as drug targets for experimentation. The derived subnetworks possess cancer-related pathways as well. The SDA-derived breast cancer subnetwork is compared with that of tools such as MCODE and Minimum Spanning Tree, and observed a higher enrichment (75%) of significant elements. Thus, the proposed nature-inspired algorithm is a novel approach to derive the optimum dys-regulated subnetwork from huge molecular network.

## 1. Introduction

Communities are significant components that are found in networks, such as social networks and biological networks. Its constituent elements are highly interconnected to perform the intended function. The structural, as well as functional, aspects of biological systems are well represented as biological networks comprising of different biomolecular elements. These networks can encode knowledge about local molecular interaction as well as some higher-level cellular communication. Studies show that changes to the network properties are very much linked to the phenotypes, such as tumors and mendelian disorders [1]. Network data in the form of interactome, functional regulatory networks and gene co-expression networks, along with other public repositories, helped biologists to gain a deep understanding of variations in cellular processes. The healthy condition of human beings can be considered the result of the perfect functioning of biological networks. While investigating the mechanism of diseases, it has been found that diverse causes of complex diseases act together to dys-regulate the same components of the cellular system [2].

Consequently, the network biology approach has emerged as an effective approach for understanding the underlying mechanism of complex diseases, including cancer [3]. In various cancer types, the disease condition is reflected through the perturbed state in pathways or molecular subnetworks [4,5]. Subnetworks are a collection of inter-connected molecules that perform a particular function. Finding dys-regulated subnetworks will help extracting useful biological information. Furthermore, mapping molecular expression data with protein interaction networks is found to be an efficient approach for effectively elucidating patterns from the network [6]. The integrative approach of combining gene expression data with other biomolecular networks was found to be efficient in extracting disease phenotypes [7]. An individual-specific network was constructed using gene expression correlations and protein–protein interactions (PPI). Here, the interacting genes in the network were found to be associated with disease states. Also, this approach could find some proteins linked to diseases that act as potential therapeutic targets [8]. In the target-centric method of drug discovery, a single target approach fails in complex disease scenario due to drug resistance and other facts [9,10].

Synergistic drug combination therapy has become a new trend, targeting pathways and modules consisting of multiple prominent targets. During the paradigm shift happening in drug discovery through systems-level target focusing, mining such pathway-based drug targets became challenging. This paper concentrates more on investigating network-oriented targets that could supplement the synergistic drugs in combating complex diseases. Thus, mining of dys-regulated subnetworks in multi-omics data has gained significance in drug design as well [11].

During the past few decades, developing methods for extracting disease-related modules in molecular data was one of the major goals in computational biology. A variety of approaches have been applied to this computationally complex problem. The greedy approach, random walk, evolutionary approach and maximum clique identification are a few well-known methods, among others. Greedy methods such as Module Analysis via Topology of Interactions and Similarity Sets (MATISSE) and DIAMOnD employ seed molecule selection followed by expansion to derive disease modules [12,13]. Starting from the seed genes, neighbouring nodes are explored based on connectivity significance [13]. Though the resulting disease modules are validated biologically, the greedy approaches fail to find optimum global networks. Another greedy method based on multivariate analysis used the scoring technique to derive a differentially expressed subnetwork [14]. As these approaches are developed based only on exploitation to construct the path, a global optimum solution is not guaranteed.

GLADIATOR is an algorithm that made use of the evolutionary global search approach to derive disease modules. A simulated annealing algorithm is applied here to maximise the gold standard module similarity measure [15]. Unlike other evolutionary algorithms, it uses a similarity index concerning known disease modules as an objective function. Moreover, it does not perform any statistical evaluation of the obtained results. However, the pure evolutionary algorithms require a precise objective function to measure actual perturbation in the module. HotNet2 is another algorithm developed for finding cancer subnetworks by mapping the connection strength to heat diffused over the network links [16]. EnrichNet is a random walk approach associated with restart ability to identify known subnetworks that are strongly connected to input genes [17].

Walktrap-GM is another algorithm that follows a random walk, exploiting the neighbours through the transition probability assessment on the weight value. A merge process of selected communities was also done to maximise the network modularity. Though this approach finds cancer-relevant modules, due to community-related computations, the complexity becomes O(n^3^) for sparse data [18]. A multi-objective approach is implemented, combining the properties, including module scores from gene expression, the pathway coverage score and connectivity measure [19]. Although prior information regarding pathway enrichment is incorporated into the algorithm to extract active modules, the drug-related functionality analysis is not provided. Breast cancer modules were generated by IODNE by running a modified minimum spanning tree algorithm upon gene-protein data. This approach has extracted dys-regulated modules with the presence of a few drug targets. However, no statistical analysis has been conducted to validate the retrieved modules [20].

In our approach, we propose an ensemble of nature-inspired greedy approaches where the algorithm complexity is reduced. Most of the existing approaches initiate the search process from genes that are found to be relevant either topologically or biologically. Moreover, these methods suffer from extensive computations in the form of the repeated objective value calculation. In the proposed algorithm, the searching is performed by multiple agents starting from random nodes in the network and hence avoids the necessity for any prioritisation of start nodes. Additionally, the algorithm complexity has been reduced over existing greedy approaches.

To test the proposed framework, gene expression data of the two most aggressive types of cancers, which affect the female and male category, were considered. Triple-negative breast cancer (TNBC) and colorectal cancer (CRC) samples were taken to generate the weighted network and for the subsequent subnetwork finding.

## 2. Materials and Methods

### 2.1. Dataset

In this work, microarray data were used for the analysis as they can be easily accessed and pre-processed quickly. The microarray data used for the analysis were downloaded from a genomic database, Gene Expression Omnibus (GEO) [21]. Moreover, efficient and easy-to-use tools are available for the processing of microarray expression data.

The TNBC Dataset includes GSE15852 (Affymetrix U133) comprising 43 tumor samples and 43 normal samples. The analysis for CRC was done with two Affymetrix microarray data sets GSE77953 and GSE113513. The first set comprises a total of 58 samples pertaining to various stages of tumor samples (17 adenoma, 17 carcinoma and 11 metastasis) along with 13 normal samples. The differential analysis needs a group of tumor samples and normal samples. However, each of these cancer stages differs in the characteristics. We took 17 carcinoma samples and 13 normal samples for the analysis. The GEO2R tool does not consider this as an unbalanced data set, as it processes the samples as tumor and normal groups. The second data set GSE113513 consists of 14 pairs of normal and tumor tissues.

### 2.2. Proposed Approach

**Figure 1:**
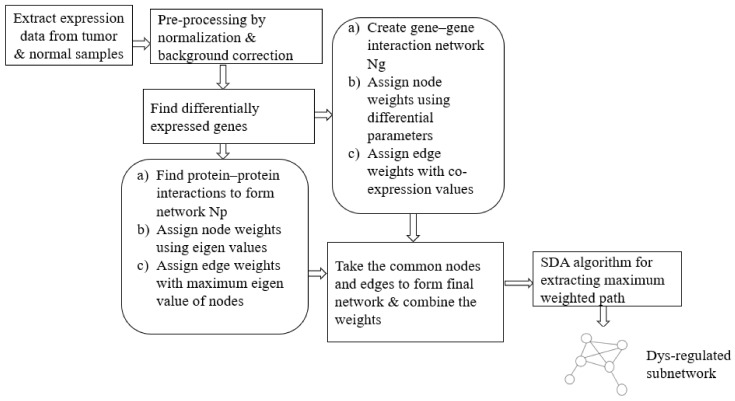
Data Flow of the proposed approach for subnetwork detection. The Smell Detection Agent (SDA) optimisation algorithm is applied on the network created using gene interaction data and protein–protein interaction data.

This work aims to extract an optimum subnetwork from an integrated network curated out of differentially expressed (DE) genes. The optimality of the subnetwork in disease condition is defined in terms of maximum dys-regulation of the molecules as well as maximum connectivity. The set of DE genes was initially extracted from the microarray gene expression data of tumor and normal samples. Then, corresponding to the DE genes, a functional correlation network and the corresponding PPI network were constructed. By making use of statistical parameters of differential expression analysis and the topological properties of the PPI network, weights were assigned for both network components. Later, the integrated network data were given as input to the heuristic Smell Detection Agent (SDA) algorithm. One major goal was to develop a less complex optimisation algorithm that can find the best possible subnetwork. Accordingly, agents of the proposed SDA algorithm explore various paths (subnetworks) using heuristic information extracted from nodes and links. The overall steps for the proposed approach are shown in Figure 1.

#### 2.2.1. Deriving DE Genes

After accessing the raw data of data set GSE15852, background correction and normalisation steps were done using the Multi-array Average (RMA) function of biocManager v12 in R language. BiocManager is a CRAN package used for installing and accessing software for the statistical analysis of genomic data.

The duplication of probes was also eliminated. The obtained data were subjected to differential analysis using the limma package [22]. Relevant functions were used to fit a linear model, generate t-statistics and necessary computations for deriving a differentially expressed gene list table.

#### 2.2.2. Curating Integrated Network

The input to the proposed SDA algorithm is the weighted network made out of the DE set of genes. This section describes how these weights are derived from different sources. As part of extracting the subnetwork with differentially expressed and highly connected genes, a network was curated from both the gene–gene interaction network and the corresponding protein interaction network. The weight assigning method followed here is an extended method used in IODNE. The integration of two weight values is expected to support and expedite the module extraction process. The significance of protein interaction data is that it provides the connection strength of genes. The functionality of proteins is regulated by their interaction. If two proteins are strongly connected, then the probability of sharing the same functionality is more. Moreover, these genes could be highly associated with disease mechanisms. Thus, using the PPI data would help expediting the extraction of genes that are strongly related and with the same functionality.

##### Gene Interaction Network (N_g_)

The first network corresponds to the functionally correlated genes in normal and tumour samples. The weight values were assigned to each node/gene based on the statistical measures of differential expression.

*g*(*i*)_*w*_ = *g*(*i*)_|*t*-*value*|_ + *g*(*i*)_|*log*(*fc*)|_ (1)

Here, *g*(*i*)_|*t*-*value*|_ is the absolute value of the *t*-*value* for *i*th gene. The combined *t*-*value* and *log*(*fc*) value was taken to assign the node weight.

As network *N_g_* reflects the functional association of genes, the gene pair correlation values were used as the link weights. The gene correlation value of (*g*_*i*_, *g*_*j*_) indicates how strongly these genes are associated with their expression values. The most popular and efficient Pearson correlation value of a gene pair is calculated using the R tool. The association coefficient values were computed for all N genes in normal samples and the tumour samples and assigned to matrices *Mcorr_nor_* and *Mcorr_tum_*. The final correlation matrix *Diff_corr_* is generated by computing the difference between these two intermediate tables as

*Diff_corr_* = *Mcorr_nor_* − *Mcorr_tum_* (2)

**Figure 2:**
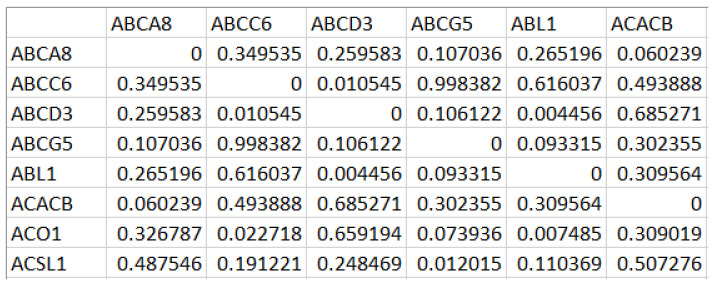
Correlation matrix generated from the Differentially Expressed (DE) gene set with rows and columns corresponding to the differentially expressed genes, and each cell holds the measure of the difference in correlation values across the samples.

A portion of *N_g_* generated for the first DE gene set is shown in Figure 2. While mapping the network *N_g_* onto the graph *G_g_*, we have computed the edge weight from the correlation value and the STRING database’s functional association score for the corresponding protein interactions [23].

##### Protein–Protein Interaction Network (N_p_)

The PPI network created was used to extract the connectivity patterns of co-expressed genes corresponding to the DE set of genes. While mapping this network *N_p_* onto the weighted graph *G_p_*, the connection strength among the proteins was also considered. The node weight and link weight were assigned considering this topology feature of proteins. Accordingly, the eigenvalue for each protein in the network was computed to extract its influence over the entire network.

As an accurate centrality measure, the eigenvector considers neighbouring nodes of the current nodes. It is described as a function of the degree of current vertex *n*_i_ and its adjacent vertices. For a given matrix A corresponding to the input graph, a scalar λ is an eigenvalue if it satisfies the condition AV = λV, and V is a non-zero vector, considered the eigenvector corresponding to λ. To represent the connection strength, different centrality measures are used. One simple approach is using degree centrality, which considers only the number of connections of the given protein in PPI network. The eigenvector is a more efficient method, which considers the connection strength of the current node as well as the connection of associated neighbours. Thus, this measure gives an accurate quantity for connection strength among proteins. The selection of the most suitable node/gene in the network is more important, as far as the subnetwork extraction is concerned. This node selection is done based on the weights assigned to the nodes. The eigenvalue is a significant part in defining weights.

Here, the protein network *N_p_* is the input matrix for eigenvalue computation. The R function was used to derive an eigenvector of size *m*, which corresponds to the number of vertices in the protein network. In contrast, while mapping *N_p_* → *G_p_*, two vectors *V_p_* for vertices and *E_p_* for edges were generated. Here, the result of eigenvalue computation *V_eig_* was assigned to *V_p_* as the weight of nodes in graph *G_p_*. Each node of *G_p_* was assigned a weight *w_i_*, where *w_i_* ϵ *V_eig_*.

To compute the edge weight in *G_p_*, the maximum score of proteins forming the edge is taken. For each edge *e_i_* *ϵ* *E_p_*, if *e_i_* is composed of (*g_k_*, *g_l_*), then

*w* (*e*_*i*_) = *max* [*w*(*g_k_*), *w*(*g_l_*)] (3)

##### Generating the Final Network (N_f_)

The final network creation has now been reduced to the weight integration process. The weights of *N_f_* will reflect both functional properties and topological properties. The size of the edge set becomes the same as the number of links in *N_p_*. The node weight becomes

*c*_1_ × *g_i_*(*w*) + *c*_2_ × *eigen_value* (4)

where *c*_1_ and *c*_2_ are tuning parameters. Here, we assigned 0.5 to assign equal weights to both the factors. Similarly, the edge weight is calculated as

**Figure 3:**
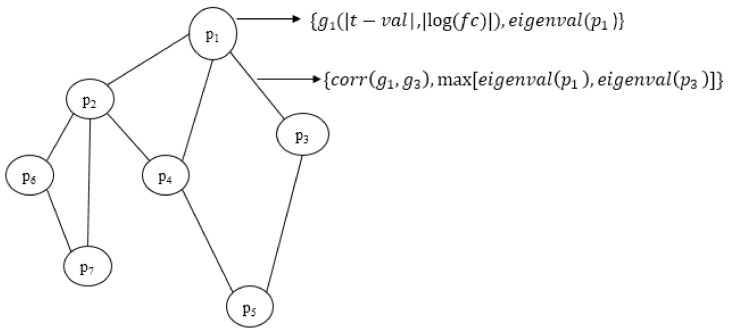
Graphical representation of the portion of the integrated network with final weights. Node weight comprises the differential weight of the gene *g_i_* and the topological weight of the corresponding protein *p_i_*. Edge weight comprises the correlation value of genes and the connectivity score of proteins in the PPI graph.

*d*_1_ × *total_linkweight* (*G_g_*) + *d*_2_ × *link weight* (*G_p_*) (5)

where *d*_1_ and *d*_2_ denote tuning parameters to adjust weight contributions. Various combinations of values between 0.1 and 0.9 were used as the tuning parameters. However, based on the results obtained, the final value pair was chosen as (0.5, 0.5). The graphical representation of the integrated network is given in Figure 3. In the figure, only the elements that are to be combined are shown. The parameters for combining the attributes are not included in the weight representation.

#### 2.2.3. SDA Algorithm

Nature-inspired algorithms have been proven as efficient in solving diverse optimisation problems, including biodata mining [24]. SDA is a recently developed optimisation algorithm suitable for path-finding applications. The algorithm mimics the behavior of dogs, described as agents, in order to detect the optimum path. Dogs are creatures known for their sniffing as well as memorising ability and therefore are trained for various target-finding applications. They also mark their region or territory by some specific biological mechanism, such as urination. Due to the superior capacity of olfactory cells, they can easily identify marked smell spots while searching paths to reach their destination. Moreover, this is a suitable mechanism that prevents other dogs from entering one’s territory. The basic SDA algorithm was developed and applied successfully for solving shortest path problems [25]. There exist plenty of optimisation problems known to be Non-deterministic Polynomial time (NP)-hard but easily solved by a nature-inspired metaheuristic approach. The basic SDA algorithm applied on the shortest path problem finds the optimum solution in a parallel search mechanism, achieved through multiple agents performing the search.

The agents (dogs), as well as smell spots (search points), constitute the algorithm environment. Initially, each agent is assigned an identification code known as a signature and a region size. These agents search for the nodes in their own territory by exploring the most suitable unmarked spot. The most suitable node is chosen based on the amount of smell value secreted by it. This exploitation terminates when an agent reaches the destination. All the agents with varying capacities are expected to find independent paths. Finally, the algorithm returns the optimised path with respect to a suitably defined objective function.

One of the most successful applications of the SDA algorithm is seen in the field of advanced computer networking. In software-defined networks (SDN), the centralised controller has applied this algorithm to find the optimal path for packets [26]. In this paper, we extended the basic algorithm to modify a few properties to provide the algorithm the ability to return the global optimum module out of the huge molecular network. In the basic algorithm, all agents start from the same start location. Additionally, the search process terminates at the destination node. In the proposed SDA algorithm, each agent will begin searching from different nodes, and the termination criterion is set as per the accepted region size. The detailed steps devised for subnetwork extraction from our integrated network are given as Algorithm 1.
**Algorithm 1** SDAInput: Weighted Network *N_f_* {*V_f_*, *E_f_*}, Module size *m*Step 1: Initialize source positions: *sr*[]
Number of agents *n_a_*Number of smell spots *n_s_* = *V_f_*, gene countStep 2: Create smell spots/nodes(i) Assign smell value by*s* = *c*_1_ × *x* + *c*_2_ × *y*, *x* and *y* are the differential and topological weight of nodes*c*_1_*andc*_2_ are tuning coefficients(ii) Mark node as ‘unvisited’Step 3: Create agents and assign start nodes to each agentFor *i* = 1 to *n_a_**st*[*a_i_*] = *sr*[*i*]Step 4: Initialize link smell as s*_l_* = *E_f_*Update smell value bys*_l_* = s*_l_* + *δ* × *p*, *δ*: smell decrement constant,*p*: accumulated weight from the current nodeStep 5: For each agent *a_i_*current node = *st* [*a_i_*]while (path size < *m*)Find neighbour list *nb*[]Choose the next node *Nx* from *nb*[]if (*Nx* = Unvisited) and (link smell is maximum)Include *Nx* into path and mark *Nx* ‘visited’Compute total path weight FStep 6: Return the maximum weight path as solution

The objective function for SDA has been defined based on both the measure of differential expression and the topological strength. These two aspects were computed and assigned as weights on nodes and edges in the network. The algorithm finally generates the optimum path, which has the maximum weight based on the following objective function

F = 1/*m* ∑*w_i_* + 1/*q* ∑*w* (*g_i_*, *g_j_*) (6)

for node count *m* and edge count *q*.

**Figure 4:**
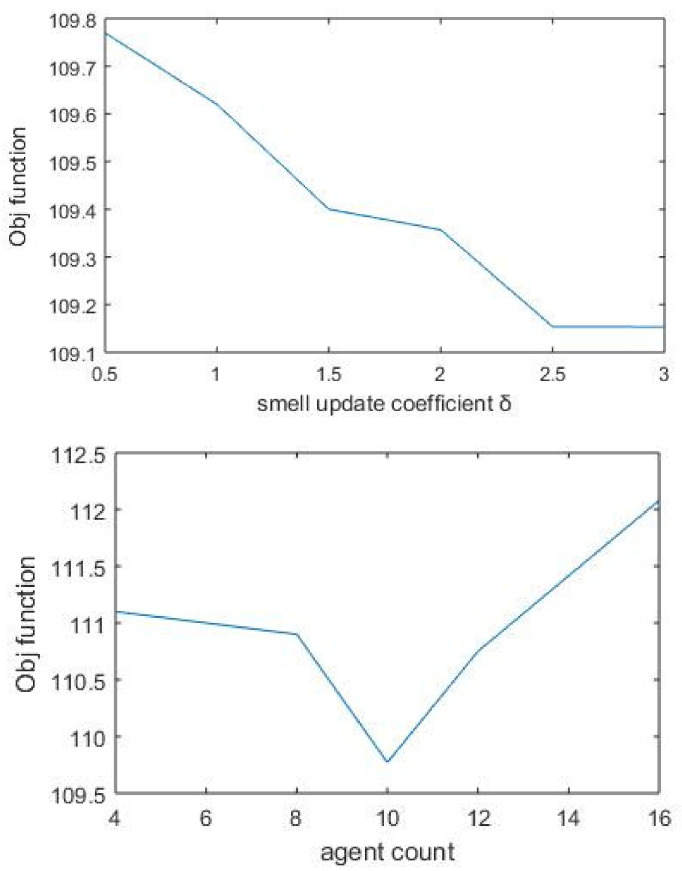
Objective function based on varying values of parameter δ. The smell update coefficient takes the value 0.5 corresponding to the maximum objective function. Agent_count represents the number of agents used by the algorithm for finding separate paths. After performing multiple runs, final value is taken as 8.

The algorithm parameter agent_count was given different values by keeping other parameters fixed. For the same agent_count value, the algorithm was run ten times with varying start points. The final value was taken by computing the average of all objective function values. Though much significant difference was not observed with objective values, the optimum performance value found was 8. The smell update coefficient was used as the proportionality coefficient when agents put smell value for protein–protein links. Unlike the basic SDA algorithm, here, δ was applied as an increment constant. Accordingly, we put different values for δ, and the value corresponding to the maximum objective value was chosen (Figure 4). As the number of nodes increases with the k value, the objective value is also increased. Finally, the robustness of the algorithm is checked by running the same process repeatedly with different parameter combinations.

#### 2.2.4. Pathway Enrichment Analysis

The association of the SDA-derived subnetwork with various functional pathways was investigated through the DAVID (Database for Annotation, Visualization and Integrated Discovery) tool [27]. It is a database which provides gene annotation as well as functional details curated by sophisticated experiments. The genes of our module were analysed by the tool so as to extract the biological processes and pathway enrichment. From the results, pathways with *p*-value < 0.05 were considered as the most significant ones in the disease.

## 3. Results

### 3.1. TNBC Data Analysis

Triple-negative breast cancer is one of the most aggressive subtypes of cancer in around 15% of the detected cancers. The absence of three receptors—estrogen, progesterone, and hormone epidermal growth factor receptor 2 (HER2)—characterises TNBC in tumor samples. The data set GSE15852 used in this study consists of samples collected from patients of different age groups [28]. After analysing the dataset with the limma package, we obtain the table with values generated for parameters *p*-value, adjacent *p*-value and log(fc) value. To filter DE genes, we have set the selection criterion as adjacent *p*-value < 0.01 and |log(fc)| ≥ 1. As the resulting gene set size was too small, the log(fc) cut-off was reduced further to 0.5. This step has helped to include more relevant genes in the derived list. Thus, we obtained a list of 1478 genes, which was used for the network construction.

#### 3.1.1. Extracting Paths

The network corresponding to the DE gene set was created by integrating gene–gene correlation data and protein interaction data. The curated network has 1478 genes and 21,320 connections. This network is applied to the SDA algorithm to extract the dys-regulated subnetwork. Here, the algorithm used different agents to find modules starting from different locations, and the maximum weighted module is designated as the optimum one. Similar to the behaviour of dogs that are reluctant to enter another one’s territory, the paths developed by the agents will also be unique. As per the size given, the algorithm derived different paths with different weights and returns the optimum one with the highest weight value indicating maximum dys-regulation of the involved elements.

#### 3.1.2. Evaluating and Comparing Algorithm Performance

This section analyses the performance of the proposed SDA algorithm in the module extraction process. Additionally, the solution quality is compared with another efficient optimisation approach known as the Artificial Bee Colony (ABC) algorithm [29]. The ABC algorithm mimics the foraging behaviour of honey bees and provides sufficient exploration ability. Thus, it provides global optimum solutions to many optimisation problems. Here, the SDA performance is measured in terms of the objective function value corresponding to various agent counts. These objective values are compared with the objective values of the ABC algorithm corresponding to different bee counts, as in Table 1.

It is seen that the number of agents required is less in SDA compared to the ABC algorithm to obtain higher objective values. Although we can increase the bee count to obtain a high-quality solution, the time complexity will also increase tremendously. To maintain the balance between the solution quality and the time requirement, we cannot increase the bee count beyond a particular limit.

Apart from this, the time complexity of both the algorithms are also compared. The ABC algorithm involves objective value computation for every iteration by each worker bee. Computing the objective value itself requires the path tracing process within the network, which is of complexity O(n^3^). Accordingly, the total complexity of the algorithm becomes N*O(n^3^) for N worker bees and a network with n nodes. Analysing the agent processes in the SDA algorithm, each agent explores the path with nlog (n) complexity. Thus, for k agents, the total complexity becomes k*nlog(n). However, as the value of k is too small compared to the value of n, it is approximated to nlog(n). Therefore, it is observed that the time complexity of the proposed SDA algorithm is less than the ABC algorithm.

The computing time of both the algorithms is also noticed and given in Table 1. It is observed that the time taken by the SDA algorithm is less than the execution time of the ABC algorithm.

**Figure 5:**
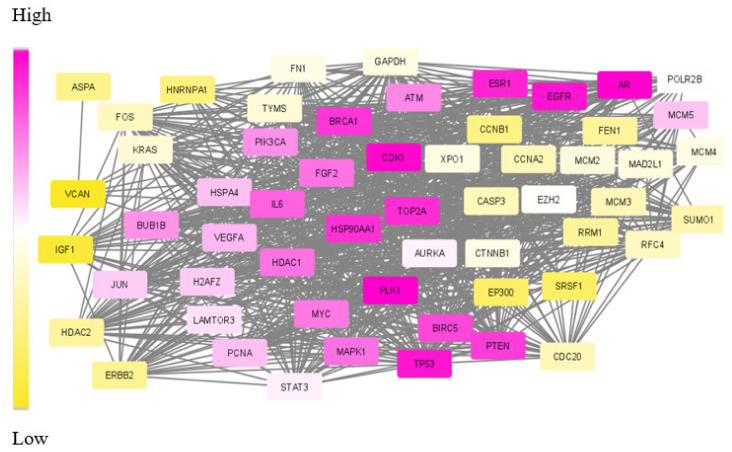
Visual representation of SDA derived dys-regulated subnetwork for Triple Negative Breast Cancer (TNBC) with 60 nodes and 940 edges. The nodes correspond to the genes in the optimum path with optimum weight values. The varying weights in increasing order is represented as colour gradient between yellow and purple.

One existing challenge in module identification problems is the non-availability of benchmark functions for evaluating the obtained modules. Existing approaches apply topological features, such as connectivity and hub nodes, to rank the resulting module. Some other tools, such as DIAMOND, make use of the similarity index regarding other disease modules. A few of them search for the existence of drug targets in the subnetwork. However, no other statistical measures were used to evaluate the obtained module. None of these methods, including disease module detecting tools, have done statistical, functional and target-related measures for analysing the module. The list of DE genes, network links and the generated subnetwork for TNBC data are given in Supplementary File S1. Figure 5 depicts the visual representation of the dys-regulated module obtained for TNBC data.

**Figure 6:**
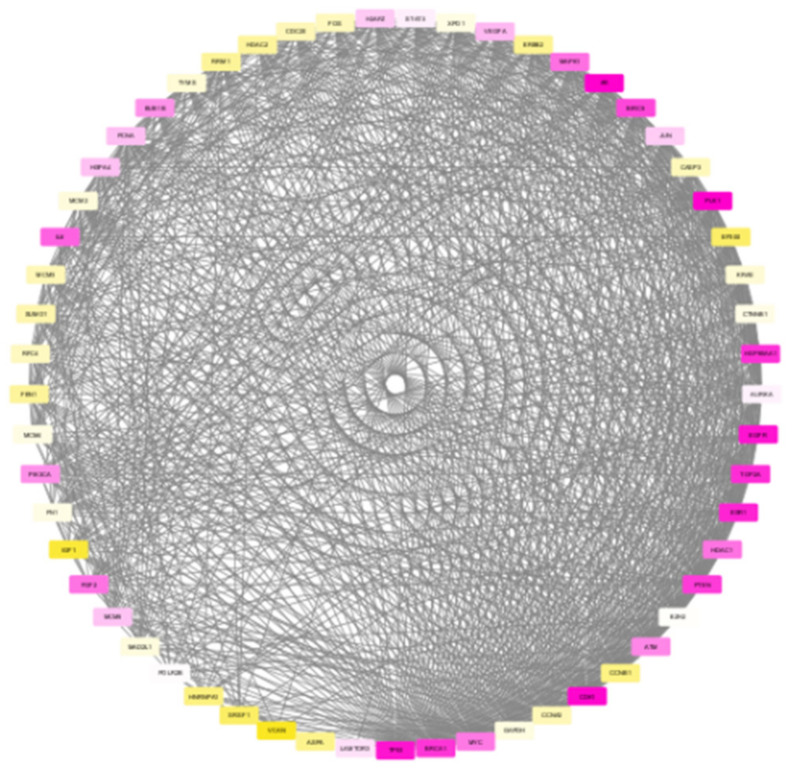
Degree-based view of the TNBC module generated by Cytoscape showing higher connectivity among the nodes.

The nodes in the subnetwork have higher weight values with respect to the differential expression and connectivity within the network. Further analysis of molecules in the subnetwork was done based on the weight values. The Cytoscape tool was used to generate the colour gradients for nodes based on the weight values [30]. Low-weight nodes are assigned yellow colour. As the weight values increase, the intensity reduces, and thus, the medium-weight nodes appear white in colour. The top nodes are assigned a purple colour. To verify the connectivity between the nodes, a degree-based view of the subnetwork is also generated as in Figure 6. By analysing this figure, it is observed that the nodes within the module are strongly interconnected. One peculiarity of the subnetwork is that the nodes are interconnected, and the degree will be high. The Cytoscape generated view shows that the nodes within the module are of a higher degree, and the nodes are strongly interconnected.

#### 3.1.3. Evaluating Biological Significance

##### Association with Disease

The proposed SDA algorithm outputs the maximally weighted module comprising many genes highly associated with TNBC. After analysing genes in the subnetwork, 80% of the genes were found to be functionally significant. *EGFR*, *TP53*, *BIRC5*, *TOP2A*, *JUN*, *BRCA1*, *IL6*, *AR*, *STAT3*, *CTNNB1*, *MYC*, *VEGFA* and *FEN1* were a few among the genes found in the module. DisGeNET is a novel platform that consists of associations between over 15,000 genes and diseases [31]. It is a rich repository of data curated from the Genome Wide Association Studies (GWAS) database and text-mined data to provide information about many complex diseases. When searched for disease-gene associations, it was found that *ESR1*, *AR*, *PIK3CA*, *CTNNB1*, *BRCA1*, *IL6*, *EGFR*, *STAT3*, *MYC*, etc., are linked to triple-negative breast cancer. Around 40% of the identified genes have a disease association score, DSA ≥ 1 in DisGeNET. Moreover, most of these genes act as biomarkers of the same disease. TNBCdb is another database that serves as a resource for TNBC by providing information on differentially regulated genes, molecular functions, and signalling pathways [32].

As shown in Table 2, 50% of the subnetwork genes were verified by DisGeNET as prognostic factors in TNBC, and 75% of the identified genes were verified by TNBCdb. We found many works in literature detecting different genes such as *BRCA1*, *PIK3CA*, *AR*, and *PTEN* as potential biomarkers of TNBC [33]. Studies show that alterations in *BRCA1* lead to dysfunction of DNA repair, checkpoint control of the cell cycle, and transcription. It also raises the risk for breast cancer and is considered one of the prominent genetic markers in TNBC [34]. Androgen Receptor (*AR*) plays a significant role in 90% of all breast cancers [35]. Another study performed on tissue microarray samples collected from 287 TNBC patients revealed AR involvement in 26% as overexpressed [36]. Similarly, tyrosine kinase receptor *EGFR* is involved in various cellular processes such as proliferation and angiogenesis. It also takes part in apoptosis inhibition by initiating a signalling cascade. A majority of TNBC samples have shown differential expression of *EGFR* and therefore treated as a potential biomarker. One noticeable point is that we obtained two candidate genes, *PIK3CA* and *PTEN*, in our derived subnetwork of TNBC. It has been shown that these two serve as cytoplasm biomarkers of TNBC with leading activities [37]. *PIK3CA* is involved in cell growth, proliferation, and cell death inhibition, leading to cancer. *PTEN* is also known as a tumour suppressor gene, inhibiting the signalling pathway lead by *PIK3CA* [38].

The significance of genes included in the SDA-derived module for TNBC data was validated with studies in literature. Disease-associated genes, biomarkers and druggable targets were identified in the subnetwork and validated with results of other methods.

##### Drug Targets

Due to the highly complex biology of TNBC samples, a thorough study became necessary in finding effective drug targets. Aiming at targeted therapy for this heterogeneous disease, more specific molecular targets are to be identified. Most of the identified biomarkers were clinically validated as promising targets. On analysing the obtained resultant subnetwork, a few already proven molecular targets were detected. We observed that the identified molecules in the derived module could act as clinically verified targets as well. *BRCA1* is one such biomarker that exists within the nucleus and is targeted by platinum drugs. *HDACs* are expression regulators playing an important role in TNBC. Effective clinical experiments are going on involving this genetic marker as a target [39]. Several clinical trials are underway targeting *AR* and sufficiently tolerated in different phases. Studies show that *STAT3* and *IL6* act as mediators for target genes *AKT* and *ERK*. Also, application of the drug Bazedoxifine seems to block *IL6* stimulated processes such as cell viability, proliferation, etc. [40]. Alterations in TopoisomeraseII alpha (*TOP2A*), commonly amplifications, were seen in different breast cancer subtypes. Moreover, it is experimentally proved that *TOP2A* acts as a predictive response to anthracycline application [41]. We found that 24% of proteins are either druggable targets or closely linked to druggable targets. The inhibiting function of *HSP90* by Simvastatin was proved to be effective against TNBC [42]. This emphasizes the role of heat shock protein 90A extracted by our module as a valid drug target. In short, 50% of the genes in the identified module are tightly associated with a disease state, and 20% of the genes are utilised in drug-related clinical experiments.

The aforementioned already proved drug targets and relevant biomarkers are in purple in the subnetwork of Figure 5. Apart from these, a few more genes were also found in the top position of our extracted module and appear in dark purple.

##### Proposed Targets

The constituent molecules of the derived subnetwork are filtered based on the gene function and weight values to be proposed as novel drug targets. Accordingly, based on the behaviour in TNBC, *ESR1* is not considered for the analysis. On analysing the weights, it is observed that the top-weighted molecules such as *TP53*, *MYC*, *JUN*, etc., are already validated biomarkers or drug targets. Therefore, a weight threshold is applied as a filter to extract molecules that are significant but not over-researched. As the weight is defined using (eigenvalue, log(fc)) pair, the threshold is fixed as 0.7 < log(fc) < 0.95 and 0.4 < eigen < 0.6. Accordingly, the genes with weight values in this range are identifies as *PLK1* (0.52, 0.79), *CTNNB1* (0.6, 0.82), *IGF1* (0.44, 0.85), *AURKA* (0.50, 0.91), *PCNA* (0.44, 0.79), *HSPA4* (0.49, 0.86) and *EP300* (0.6, 0.79). Apart from these, *GAPDH* is also proposed as it has higher weights (0.85, 0.86) but not explored much. All these molecules can be subjected to further analysis for consideration as targets.

Searching for the applicability of these molecules as drug targets, it is seen that a few studies are conducted involving some of these genes as drug targets. *PLK1* is a gene that is suggested through siRNA-mediated knockdown screening [43]. *IGF1* is found to be a part of a signalling pathway which promotes growth of TNBC cells [44]. Thus, it is a novel candidate for the TNBC drug target.

##### Targets of Synergistic Drugs

As complex diseases such as cancer are caused by multiple proteins, a combination of drugs would help combat diseases effectively. Searching for such proteins that act as targets for synergistic drugs was one of our motives. Accordingly, we searched for the potential of proteins present in our derived module for TNBC during analysis. It has been observed that an in silico study involving synergistic drugs action against certain target proteins revealed the efficacy of those drugs on multiple target proteins in TNBC tissues. The combination of afatinib and YM155 exhibited a synergistic cytotoxic effect across multiple TNBC models by inhibiting *BIRC5* and *EGFR* proteins [45]. Our module also has these two proteins *BIRC5* and *EGFR* that can be denoted as synergistic targets. Additionally, the proteins which are identified as targets can be analysed further to showcase synergistic effects of associated drugs.

##### Pathways Identified

The derived subnetwork is expected to contain genes that belong to some significant functional pathways. Accordingly, the obtained TNBC module genes were submitted to the KEGG tool. It has returned 70 pathways contributing to various cellular and other functionalities. We have evaluated the genes involved in those pathways with a *p*-value < 0.05 and observed that 82% of the module genes span over these KEGG pathways. Table 3 shows a few pathways comprising module genes and found relevant in cancer progression and other cellular processes.

After obtaining the enrichment of disease-relevant elements within the SDA-derived subnetwork, a comparison is done with enrichment in other methods. The presence of relevant drug targets, biomarkers and pathways in the TNBC module is compared with that of IODNE, as well as MCODE [20,46]. MCODE is a tool developed based on a clustering technique and returns multiple modules with varying scores. IODNE was developed using the Minimum Spanning Tree technique for extracting modules from networks of breast cancer data. We have analysed the results of these techniques, and the estimate taken is shown in Table 4.

It is observed that SDA-derived module has the highest enrichment compared to the modules of other techniques. This observation proves the superiority of the proposed approach in deriving subnetworks from biological networks.

#### 3.1.4. Statistical Assessment

Considering the quality of the obtained modules, a statistical evaluation is as important as biological validation. Here, we have used the modularity index, known as “local modularity,” as one evaluation criterion [47].

In the graph corresponding to our generated network *N_f_*, we know all the connection details of a small portion S (subgraph), and for the remaining portion S’ in G, we know only nodes that are adjacent to S. Consider those nodes in S that are connected to at least one node in S’, then these nodes said to constitute a boundary for S. This boundary B is said to be sharp if it has a smaller number of connections to S’ but more connections with nodes in the community S. In such a context, local modularity *R* is defined as

*R* = ∑(*B_ij_*
*δ*(*i*,*j*))/∑*B_ij_* (7)

Here, *δ*(*i*,*j*) becomes 1 if there exists a link between *B* and S; otherwise, it will be 0. As per the definition, to keep the best modularity, the *R*-value is expected to be low. We computed *R* for the obtained module, and it is 0.17, which is low. This indicates the quality of the derived module.

### 3.2. CRC Data Analysis

CRC is another type of cancer that leads to a higher death rate among men of a particular age group [48]. Initiated as adenoma, this disease may develop into a metastasis condition with adverse effects on other organs [49]. As with other cancer types, CRC is also treated with targeted therapy, prepared for affecting predominant markers, such as *VEGFA* and *EGFR* [50]. Here, we have used two data sets, GSE77953 and GSE113513, for analysis [51]. After the normalisation process by the limma package, differentially expressed genes were extracted using GEO2R. After we applied the criteria *p*-value < 0.01 and |log(fc)| > 1, we obtained two DE gene lists S_1_ and S_2_ comprising 1945 genes and 1748 genes, respectively. Then we have extracted common genes of these two lists and obtained a list of 245 relevant ones. However, our aim is to construct a network of genes with functional and topological significance. Therefore, we have extracted a subset of genes with |log(fc)| > 1.5 from both S_1_ and S_2_. These genes were combined with common 245 genes, and finally, a DE list of 825 genes was curated.

The network was constructed, and weight was assigned based on the gene interaction values and topological scores. The curated network has 825 nodes and 7127 links. Then SDA algorithm with random start nodes was applied to this network. The extracted subnetwork was visualised using Cytoscape. The resultant optimum module consists of 60 nodes and 666 edges, and the relevance of each molecule was investigated. The DE gene list, edge list of network and the output genes in the generated module are provided in Supplementary File S2.

**Figure 7:**
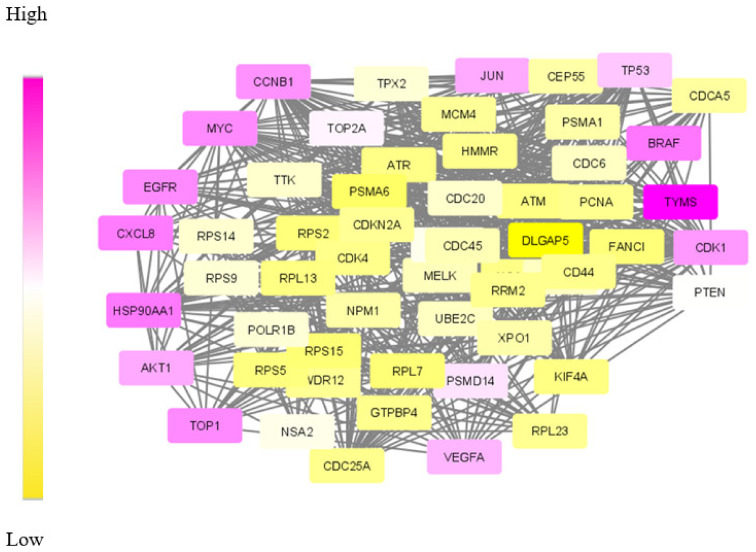
The optimum dys-regulated subnetwork generated by the SDA algorithm for CRC. The yellow nodes represent genes with low weights in the module and top-weighted nodes are purple.

Validating result: The data of differentially expressed genes itself is found to be significant for understanding the mechanism of CRC. This is due to the lack of enough molecular data in the form of targets and dys-regulated genes. Therefore, the obtained set of DE genes was compared with the gene list compiled by other methods. Among the common 245 genes, 90% of genes were matched with the results of the mRmR (maximum relevance minimum redundancy) method and the Human Protein Atlas database [52,53]. The dys-regulated subnetwork for CRC was extracted using the SDA algorithm run with seven agents. The obtained module is shown in Figure 7.

To evaluate the significance of genes/proteins in the obtained module, we have considered a few techniques in literature, and the findings in comparison are given in Table 5. A bioinformatics analysis was done on CRC gene expression data using existing tools, and a dense module of candidate genes was obtained by Chen et al. [54]. Among the 16 genes found within this module, extracted by Cytoscape, 11 genes were overlapped with genes found by our approach. As one of our criteria for deriving dys-regulated module was maximally connected module, this high number of overlapped genes (78%) indicates the relevance of our obtained module in terms of connectivity. Additionally, the hub genes identified by this method overlap with the module genes in the SDA algorithm. The underlying molecular mechanism of most cases of colorectal cancer has been proved to be associated with genes such as *KRAS*, *APC*, *TP53*, *EGFR*, etc. [55]. Our extracted subnetwork contains most of these genes, including *TP53*, *BRAF*, *PTEN*, *EGFR* and *APC* variants.

#### 3.2.1. Biomarkers and Drug Targets

The most promising fact noticed in the results is approved drug targets in the obtained module. A total of 15 most prominent Food and Drug Administration (FDA)-approved drug targets along with the associated drugs were presented in an ontology-based network analysis approach [56]. Among these target genes, five are present in the higher weighted genes of the SDA-derived subnetwork. Overexpression of *CDC20* was proved to be associated with a prognostic marker for colorectal cancer [57]. A recent study reveals the scope for further clinical studies to consider a well-known tumour-related gene MYC as an effective drug target. Based on the experimental data, it was suggested that inhibiting c-MYC expression may stop tumour growth. Its downstream target genes also act as effective targets for tumours therapy [58]. *CDK1*, *MAD2L1*, *MYC* and *CCNB1* were also proposed as biomarkers as they associate with cell cycling-related pathways [59].

#### 3.2.2. Proposed Targets

The gradient colouring given to the module nodes based on the weights makes higher weighted nodes appear in purple. Additionally, the analysis of module genes shows that the relevant molecules that are identified as drug targets and biomarkers are purple. Accordingly, molecules denoting the top-weighted nodes that appear in purple in Figure 7, and *AKT1*, *CCNB1*, *HSP90AA1* and *CXCL8* are proposed as drug targets for further analysis.

#### 3.2.3. Pathways Identified

The KEGG database returned a set of pathways enriched with the module genes of CRC. It is found that these pathways are related to cell cycle progression, cancer-related function or signalling processes. Table 6 shows the significant pathways observed for CRC along with their functionality and involved genes. One of the obtained pathways represents the colorectal cancer pathway consisting of genes *JUN*, *MYC*, *AKT1*, *BRAF* and *TP53*. This result proves the fact that the derived dys-regulated subnetwork has a tight association with disease, and the genes are relevant in other biological processes as well.

By analysing the genes present, it is observed that 80% of the module genes are present in the functionally relevant pathways. Thus, it is evident that our proposed algorithm is capable of extracting the significant module in CRC data.

## 4. Limitations and Future Work

Our proposed approach has succeeded in extracting the de-regulated subnetwork in both TNBC and CRC data. In TNBC data, we could detect relevant target proteins, including proteins for synergistic drugs. While analysing the CRC module, we could find a couple of disease biomarkers and drug targets. However, in the data modules identified, our approach failed to identify some particular marker genes. KRAS and PAICS are two significant genes in CRC, but these were not included in the derived module. This may be due to the limited number of data samples taken for analysis.

Furthermore, we have considered microarray expression data for the analysis. With the advancements in sequencing technologies, RNA-Seq data sets are currently available for analysis. We could not use these transcriptome data for this study due to some technical constraints. However, our future work would concentrate on extracting RNA-seq data of cancer samples so as to derive more accurate results.

Similarly, the limitation with the smaller number of samples would be overcome by extracting more data samples. Additionally, the differential expression analysis would be performed by highly sophisticated methods. The final DE gene list would be prepared by taking the common genes obtained by each data sample. This is expected to improve the confidence of the initial seed genes for further analysis.

Another aspect of the de-regulated module that can be considered is the copy number variation count. Combining these three attributes would make the tool much more effective in module extraction.

## 5. Conclusions

We have proposed an optimisation framework to elucidate the dys-regulated subnetwork from a weighted network curated out of differentially expressed genes and the corresponding proteins. An efficient nature-inspired SDA algorithm was designed for this path extraction. The most promising feature of this algorithm was the reduced time complexity of n (log n) for n number of nodes in the network. This algorithm has successfully derived the most optimum set of nodes and links based on the topological and differential expression scores. As we provided multiple agents, the algorithm has chosen the best path as the final result. These nodes were mapped to genes/proteins to form the molecular subnetwork to extract maximum biological information. Once we can extract such modules, we can process it further to mine useful information.

The biological evaluation of the obtained genes in the module has revealed the efficacy of our proposed approach. Due to the deadly nature and higher death rates, we have chosen TNBC and CRC data sets for analysis. Overall, in both these cancer types, 70% of the genes were biologically validated, including drug target prediction. In CRC, we proposed new drug targets considering the significance of the genes in the derived module.

Compared to the other approaches, the major advantage is that a single algorithm is sufficient to elucidate the module comprising of biomarkers, hub genes, drug targets, and other aspects. In most of the existing approaches, multiple tools and techniques are required to obtain all this information.

Above all, these modules’ future applications can be further analysed to access synergistic drug targets for the concerned disease. Through effective mechanisms, the synergistic targets which are likely to be bound by multiple drugs or small molecules can be recognised.

## Figures and Tables

**Table 1:** Comparing performance of the proposed algorithm and Artificial Bee Colony (ABC) algorithm.

SDA Algorithm		
No. of Agents	Objective Value	Time (s)
4	111.1	0.03
8	110.98	0.05
12	111.0	0.07
14	111.5	0.08
**ABC Algorithm**		
**No. of Bees**	**Objective Value**	**Time (s)**
20	104.37	0.238
30	105.18	0.467
40	108.94	0.574

**Table 2:** Module gene associations with diseases for TNBC gene set, verified with other methods.

Molecules Approved/Undergoing Studies	Significance Observed	Genes in SDA Module Overlapped with Other Methods	Number of Overlapped Molecules
*BRCA1*, *BRCA2*, *EGFR*, *PIK3CA*, *AR*, *PARP*, *PD1*, *PDL1*, *TP53*, *FGFR*, *VEGF*, *TROP2*, *NOTCH* [34,36,37]	Biomarker	*BRCA1*, *EGFR*, *PIK3CA*, *AR*, *PTEN*, *VEGFA*, *TP53*	7
*VEGF*, *EGFR*, *FGFR*, *PD1*, *AR*, *CTLA4*, *AMPK*, *MDM2*, *MTDH*, *ATR*, *CHK1*, *WEE1*, *HSP90*, *CDC25*, *BRCA1*, *IGF1*, *AKT*, *PIK3CA*, *PTEN*, *PARP*, *CDK4*, *CDK1*, *STAT3*, *IL6*, *TOP2A* [39,40,41,42]	Drug targets under clinical validation/pre-clinical evaluation	*CDK4*, *CDK1*, *PTEN*, *AR*, *PIK3CA*, *TOP2A*, *STAT3*, *IL6*, *BRCA1*, *HSP90*, *VEGFA*, *IGF1*	12
*PLK1*, *CTNNB1*, *IGF1*, *AURKA*, *PCNA*, *HSPA4*, *EP300*	Proposed targets	Chosen based on weights	
Genes found in DisGeNET database	Disease associated genes	*AR*, *PIK3CA*, *CTNNB1*, *BRCA1*, *IL6*, *EGFR*, *STAT3*, *MYC*, etc.	32
Genes found in TNBCdb database	Disease associated genes		45

**Table 3:** Top five pathways identified by KEGG tool from the TNBC subnetwork.

Pathway Description	*p*-Value	Genes Present
hsa04110: Cell cycle	3.84 × 10^−17^	*HDAC1*, *BUB1B*, *CCNA2*, *CDC20*, *CCNB1*, *MYC*, *MCM3*, *CDK1*, *MCM4*, *EP300*, *MCM5*, *ATM*, *TP53*, *MCM2*, *MAD2L1*
hsa05200: Pathways in cancer	5.49 × 10^−16^	*HDAC1*, *PTEN*, *FGF1*, *EGFR*, *MYC*, *CASP3*, *TP53*, *MAPK1*, *EP300*, *JUN*, *HSP90AA1*, *STAT3*, *FN1*, *IGF1*, *FOS*, *VEGFA*, *AR*, *IL6*, *PIK3CA*, *BIRC5*, *CTNNB1*, *KRAS*
hsa04115: p53signalling pathway	5.18 × 10^−8^	*CCNB1*, *RRM2*, *CDK4*, *CASP3*, *PTEN*, *CDK1*, *ATM*, *TP53*, *IGF1*
hsa04915: Estrogen signalling pathway	1.35 × 10^−^^5^	*HSP90AA1*, *JUN*, *PIK3CA*, *MAPK1*, *KRAS*, *FOS*, *ESR1*, *EGFR*
hsa05202: Transcriptional mis regulation in cancer	0.0022	*IL6*, *HDAC1*, *MYC*, *ATM*, *IGF1*, *TP53*

Pathway enrichment analysis of genes found in the TNBC subnetwork was conducted. For a cut-off *p*-value < 0.05, 55 functionally relevant pathways were obtained, and five are shown here. The list of all pathways is given as Supplementary File S3.

**Table 4:** Comparing enrichment of significant elements in the subnetwork.

Method	Path Size	Disease Genes (%)	Drug Targets	Significant Pathways	Biomarkers
MCODE	88	32 (36%)	7	4	9
MST	58	37 (64%)	10	2	7
SDA	60	45 (75%)	10	7	12

**Table 5:** Module gene associations with diseases for the CRC gene set.

Gene Symbol Identified by Other Methods	Significance Observed/Method Used	Genes in SDA Module	No. of Overlapped Genes
*TOP2A*, *CDK1*, *ECT2*, *FEN1*, *NEK2*, *BUB1B*, *RRM2*, *NCAPG*, *MELK*, *AURKA*, *CCNB1*, *DLGAP5*, *FANCI*, *CKS2*, *CEP55*, *CKAP2*	Dense module/Cytoscape [54]	*TOP2A*, *CDK1*, *FEN1*, *NCAPG*, *MELK*, *RRM2*, *AURKA*, *CCNB1*, *CEP55*, *FANCI*, *DLGAP5*	11
*TOP2A*, *PAICS*, *CDK1*, *CKS2*, *CKAP2*, *CEP55*, *VEGFA*, *NEK2*, *PHLPP2*, *RRM2*	Hub genes [54]	*TOP2A*, *CDK1*, *CEP55*, *VEGFA*, *NEK2*, *RRM2*	6
*BRAF*, *RAS*, *APC*, *TP53*, *EGFR*, *PTEN*, *SMAD4*, *MSH2*, *MSH6*, *MLH1*	Common onco genes and tumor suppressor genes [55]	*BRAF*, *TP53*, *EGFR*, *PTEN*, *APC*	
*BRAF*, *C1QA*, *C1QB*, *VEGFA*, *FCG1A*, *FCGR2A*, *FCGR2B*, *TYMS*, *EGFR*, *TOP1*, *DDR2*, *EPHA2*, *FGFR1*, *RET*, *TEK*	Drug targets [56]	*BRAF*, *TYMS*, *VEGFA*, *EGFR*, *TOP1*	5
-	Proposed targets	*AKT1*, *CCNB1*, *HSP90AA1*, *JUN*, *CXCL8*	

Relevance of genes found in the SDA-derived module for CRC data was assessed. By comparing with results by other tools, hub genes, dense module genes and drug targets were identified. Overall, 80% of genes in subnetwork was found to be validated with the compared techniques.

**Table 6:** Pathways observed during analysis of CRC subnetwork genes. This table shows a few top pathways associated to cellular functions, signalling and cancer-related processing.

Pathway Description	*p*-Value	Genes Present
hsa04110: Cell cycle	1.89 × 10^−14^	*PCNA*, *CDKN2A*, *TTK*, *CDC6*, *CDC25A*, *CDC20*, *CCNB1*, *CDK4*, *MYC*, *CDK1*, *MCM4*, *ATM*, *TP53*, *ATR*
hsa04115: p53 signaling pathway	8.55 × 10^−9^	*CCNB1*, *RRM2*, *CDKN2A*, *CDK4*, *PTEN*, *CDK1*, *ATM*, *TP53*, *ATR*
hsa03010: ribosome	2.45 × 10^−5^	*RPS15*, *RPS14*, *RPS9*, *RPS5*, *RPL23*, *RPL13*, *RPS2*, *RPL7*
hsa05200: Pathways in cancer	3.20 × 10^−5^	*HSP90AA1*, *JUN*, *CXCL8*, *CDKN2A*, *CDK4*, *MYC*, *PTEN*, *AKT1*, *BRAF*, *TP53*, *EGFR*, *VEGFA*
hsa05210: Colorectal cancer	6.76 × 10^−4^	*JUN*, *MYC*, *AKT1*, *BRAF*, *TP53*
hsa04151: PI3K-Akt signaling pathway	0.0065	*HSP90AA1*, *CDK4*, *MYC*, *PTEN*, *AKT1*, *TP53*, *EGFR*, *VEGFA*
hsa0401: MAPK signaling pathway	0.0238	*JUN*, *MYC*, *AKT1*, *BRAF*, *TP53*, *EGFR*

The pathway enrichment analysis by KEGG has returned 35 pathways for a cut-off *p*-value < 0.05. This table shows seven functionally relevant pathways comprising the top genes of the derived subnetwork. The list of all pathways is given as Supplementary File S3.

## Data Availability

Not applicable.

## Supplementary Materials

The following are available online at https://www.mdpi.com/article/10.3390/biom12010037/s1, Supplementary File S1: Input network and subnetwork for TNBC, Supplementary File S2: Input network and subnetwork for CRC, Supplementary File S3: Pathways in subnetworks.
